# Obstructive Jaundice as the Initial Manifestation of Gastric Adenocarcinoma

**DOI:** 10.7759/cureus.32478

**Published:** 2022-12-13

**Authors:** Shehbaz M Ansari, Dhrumil Patel, Yashant Aswani, Abhishek Bairy, Hiba Narvel

**Affiliations:** 1 Diagnostic Radiology, Rush University Medical Center, Chicago, USA; 2 Radiology, Seth Gordhandas Sunderdas Medical College, King Edward Memorial Hospital, Mumbai, IND; 3 Radiology, University of Iowa Hospitals and Clinics, Iowa City, USA; 4 Internal Medicine, Albert Einstein College of Medicine, Jacobi Medical Center, New York, USA

**Keywords:** contrast-enhanced computed tomography (cect), spread across the gastroduodenal junction, malignant biliary obstruction, jaundice cholestatic, carcinoma stomach

## Abstract

A 52-year-old female presented with epigastric pain, yellowing of the sclera, and vomiting for three weeks. Laboratory investigations revealed markedly elevated serum bilirubin and alkaline phosphatase levels, accompanied by a modest rise in transaminases. A clinical diagnosis of obstructive jaundice was established. Ultrasound of the abdomen depicted a mass in the region of the head of the pancreas. Contrast-enhanced computed tomography (CECT) of the abdomen revealed an infiltrative gastric mass spreading across the gastroduodenal junction and involving the ampulla of Vater. Owing to comorbid conditions and widespread lymphadenopathy, a palliative gastrojejunostomy with excision biopsy was performed. Histopathology confirmed an undifferentiated gastric adenocarcinoma.

## Introduction

Jaundice, particularly in adults, can be a marker of an ominous underlying pathology [1]. The preliminary evaluation is based on history and physical examination. Laboratory investigations such as fractionated bilirubin, alkaline phosphatase, and transaminases help classify jaundice into prehepatic, hepatic, or posthepatic subtypes [1]. Posthepatic jaundice, also known as obstructive jaundice, is further evaluated by ultrasound of the abdomen to identify the site of obstruction as intrahepatic versus extrahepatic [1]. Although gallstones are the most common cause of obstructive jaundice [1], other etiologies include cholangitis, pancreatitis, and tumors [1]. Malignant biliary obstruction is usually secondary to pancreatic ductal adenocarcinoma or cholangiocarcinoma [2]. Ampullary carcinoma, primary duodenal adenocarcinoma, and pancreatic neuroendocrine tumors comprise less common etiologies [2]. Gastric adenocarcinoma presenting as obstructive jaundice is rare [3], frequently secondary to the mass effect of metastatic periportal lymph nodes rather than direct invasion [4].

## Case presentation

A 52-year-old hypertensive and diabetic female presented with dull aching epigastric pain, vomiting, and yellowish discoloration of the sclera for three weeks. There was a history of weight loss, loss of appetite, and fatigue. There was no fever, constipation, hematemesis, or melena. The past surgical history was noncontributory. She was on tablet glimepiride and metformin for diabetes and telmisartan and metoprolol for hypertension. On examination, she was afebrile, and her vital signs were stable. She had pallor and icterus. Per abdomen examination elicited mild tenderness in the epigastrium.

**Figure 1:**
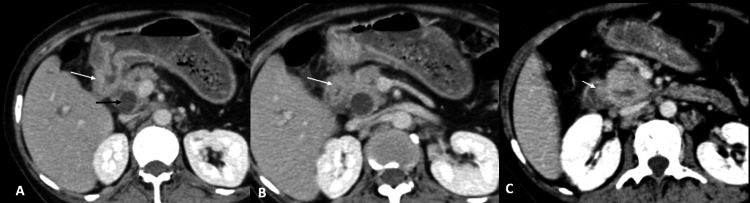
Gastric adenocarcinoma Axial sections of the CECT of the abdomen show mucosal thickening involving the antrum, duodenum, and ampulla (white arrows in Figure 1A, Figure 1B, and Figure 1C, respectively) with loss of mural stratification. CECT: contrast-enhanced computed tomography

**Figure 2:**
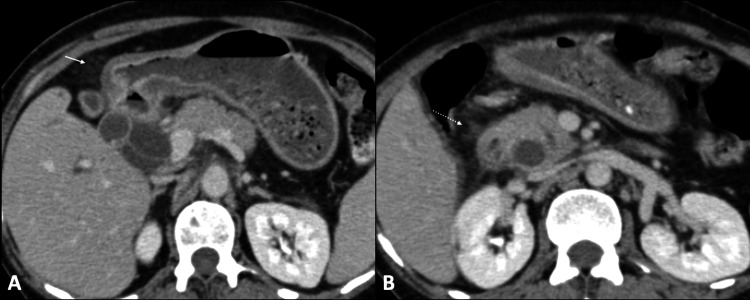
Peritoneal spread Axial sections of the CECT of the abdomen demonstrate stranding of gastrocolic (white arrow in Figure 2A) and hepatoduodenal (white arrow in Figure 2B) ligaments. CECT: contrast-enhanced computed tomography

**Figure 3:**
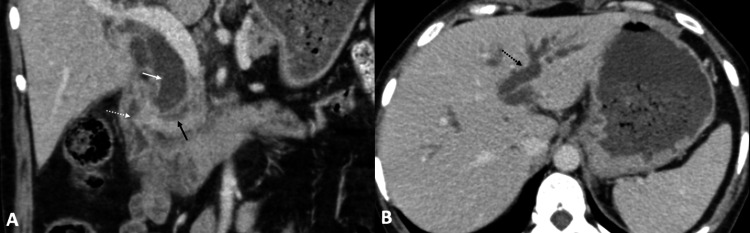
Ampullary involvement Coronal (Figure 3A) and axial (Figure 3B) sections of the CECT of the abdomen show a periampullary mass (dashed white arrow) with dilation of the CBD (white arrow) and pancreatic duct (black arrow). The intrahepatic biliary radicals are dilated as well (dashed black arrow in Figure 3B). CECT: contrast-enhanced computed tomography, CBD: common bile duct

**Figure 4:**
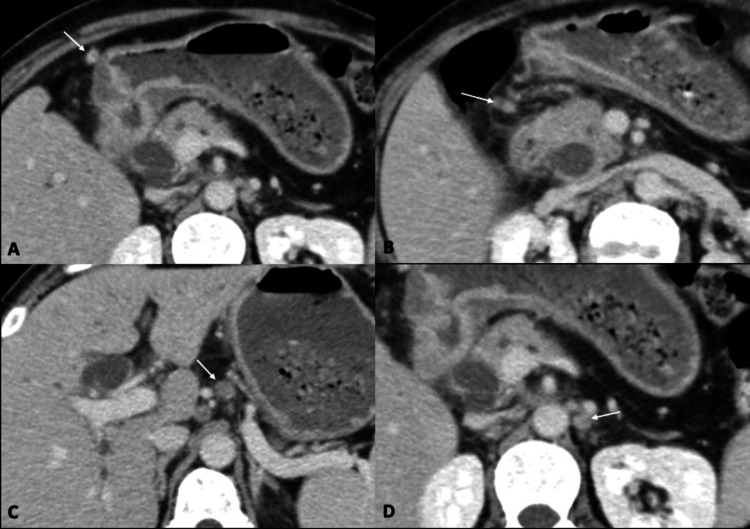
Lymph nodal spread Axial sections of the CECT of the abdomen show enhancing lymph nodes in the greater curvature (A), infrapyloric (B), celiac (C), and para-aortic (D) regions. Interestingly, except for the greater curvature, all other stations show clusters of lymph nodes. CECT: contrast-enhanced computed tomography

A complete blood count revealed hemoglobin (Hb) of 7.2 mg/dL, leukocyte count of 8,900/mm^3^, total bilirubin of 17.6 mg/dL, direct bilirubin of 14.6 mg/dL, alkaline phosphatase of 937 U/L, aspartate transaminase of 80 U/L, and alanine transaminase of 62 U/L. Hence, a clinical diagnosis of obstructive jaundice was established. Ultrasound of the abdomen revealed an ill-defined, hypoechoic mass in the region of the head of the pancreas causing marked dilation of the common bile duct (CBD) and intrahepatic bile ducts. Further evaluation with contrast-enhanced computed tomography (CECT) of the abdomen showed circumferential thickening of the antrum of the stomach with loss of mural stratification (Figure 1). There was no gastric dilation to suggest gastric outlet obstruction. The thickening extended along the D1 and D2 segments of the duodenum and involved the ampullary region (Figure 1). Additionally, there was fat stranding along gastrocolic and hepatoduodenal ligaments concerning infiltration (Figure 2). However, no diffuse peritoneal disease was identified. Both CBD and the main pancreatic duct were dilated with an abrupt cutoff at the ampulla (Figure 3). Further, numerous enlarged, round, enhancing locoregional (infrapyloric, greater curvature, celiac, and common hepatic) and metastatic (superior mesenteric and para-aortic) lymph nodes were identified (Figure 4). There was no direct invasion of the adjacent organs or peritoneal deposits or vascular involvement. No focal lesion was identified in the liver, lung bases, or bones. The circumferential thickening of the pylorus and duodenum with perigastric lymphadenopathy favored gastric malignancy over a periampullary tumor. Due to the selective involvement of gastric antrum with the sparing of the fundus and body and the presence of perigastric disease, gastric adenocarcinoma was a more likely diagnosis than gastric lymphoma.

Gastric wall thickening extending up to the ampulla was confirmed on upper gastrointestinal (GI) endoscopy. Biopsy samples were taken from multiple sites. The CBD could not be negotiated for stenting. A nasojejunal (NJ) tube was placed for feeds. For establishing a baseline level, tumor markers were sent, which showed elevated carcinoembryonic antigen (CEA).

During the hospital stay, the patient developed a fever with a leukocyte count of 21,700/mm^3^. Due to a prior failed attempt at endoscopic retrograde cholangiopancreatography (ERCP), a percutaneous transhepatic biliary drainage (PTBD) was performed to relieve biliary obstruction potentially leading to cholangitis. The fever settled over the next 10 days. Due to extensive locoregional and metastatic lymphadenopathy, a palliative procedure was planned. She underwent a Roux-en-Y gastrojejunostomy (antecolic, isoperistaltic) with side-to-side jejunojejunostomy 12 days after the admission. A choledochojejunostomy was technically challenging to perform. The postoperative period was uneventful. The patient was started on nasojejunal (NJ) feeds on postoperative day (POD) 2, which was changed to oral feeds on POD 4. The patient tolerated oral feeds well. Hence, the NJ tube was removed on POD 7. PTBD internalization was attempted on POD 9 but failed. Subsequently, the patient was discharged to follow up in the oncology clinic along with the biopsy result.

**Figure 5:**
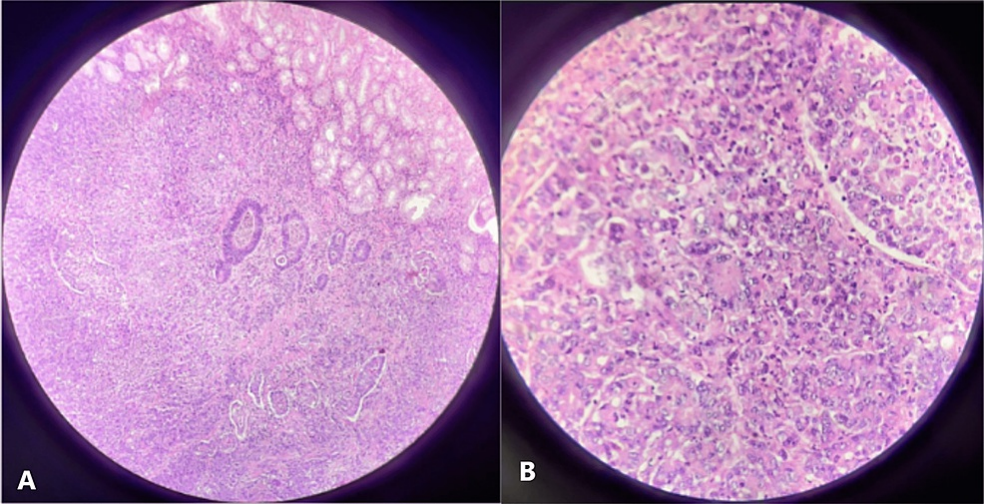
Histopathology Histopathology sections show poorly differentiated adenocarcinoma with focal glandular architecture involving the submucosa of the duodenum (A) (H&E: 200×) and sheets of poorly differentiated carcinoma cells with moderate eosinophilic cytoplasm, pleomorphic and vesicular nuclei, and prominent nucleoli (B) (H&E: 400×). H&E: hematoxylin and eosin

Histopathology of the surgical specimen revealed a poorly differentiated adenocarcinoma along with few signet cells as well as focal glandular architecture (Figure 5). The adjacent mucosa showed the presence of tumor emboli. The malignant cells nested around the duodenal papilla. Since the tumor was unresectable, palliative chemotherapy was offered. However, the patient refused further active management and succumbed to her illness two months later.

## Discussion

Gastric cancer ranks sixth among all new cancer cases worldwide and is the second most common cause of cancer-related deaths [5]. Gastric adenocarcinoma is more common in Japan, Eastern Asia, South America, and Eastern Europe [3]. It results from an interplay of genetic, dietary, and environmental factors [3].

Gastric carcinoma usually presents with nonspecific symptoms such as dyspepsia, which often delays the diagnosis [3]. Common presenting symptoms are anorexia, weight loss, and vague abdominal pain [3]. Giant tumors obstructing the gastric lumen may present with nausea, vomiting, and early satiety [3]. Presentation as obstructive jaundice is rare.

Upper gastrointestinal endoscopy with endoscopic ultrasound (EUS) is the preferred initial modality for the diagnosis of gastric carcinoma due to high sensitivity and accessibility to tissue sampling in a single visit [6]. For tumor (T) and node (N) staging of gastric carcinoma, EUS shows better sensitivity, while the CECT scan has better specificity [7]. Positron emission tomography (PET) is useful in detecting distant metastasis [8], while magnetic resonance Imaging does not have a significant role in the management [7].

The imaging findings of gastric adenocarcinoma on CECT include abnormal gastric wall thickening with enhancement and loss of mural stratification. Perigastric tumoral invasion is seen as fat stranding that may also be secondary to inflammation or desmoplastic reaction. The involvement of perigastric lymph nodes is suggested by an increase in size, round contour, irregular margins, enhancement, and the presence of a cluster of nodes [8].

Dissemination of gastric carcinoma occurs via lymphatic, hematogenous, direct invasion, transperitoneal, and subperitoneal routes [8]. Interestingly, according to the American Joint Committee on Cancer (AJCC) TNM-8 classification, contiguous involvement of the esophagus and duodenum is not considered under the direct invasion of adjacent organs and thus does not upstage the disease [9]. The AJCC and the Japanese Gastric Cancer Association divide lymph nodes as regional (perigastric and other) or distant [8-10]. Further, the N stage of the disease is based on the number of involved regional lymph nodes rather than their exact location, provided no distant lymph node is affected. The involvement of distant lymph nodes constitutes metastasis. In our case, enlarged greater curvature and infrapyloric lymph nodes comprised “perigastric regional” nodes, celiac and common hepatic artery lymph nodes constituted “other regional” nodes, and superior mesenteric vein and para-aortic region lymph nodes constituted “distant” lymph nodes.

Lee et al. reviewed 54 patients with metastatic gastric adenocarcinoma presenting with obstructive jaundice [4]. The authors concluded that the cause of obstructive jaundice was metastatic lymphadenopathy in the hepatoduodenal ligament in 50 patients, while the remaining four (7.4%) had a direct invasion of the distal CBD and duodenum by the primary or recurrent disease. Similarly, obstructive jaundice in gastric adenocarcinoma has been reported to be caused by various obstructing lesions as enumerated in Table 1 [11-17]. Overall, very few cases of gastric carcinoma presenting as obstructive jaundice due to contiguous spread along the duodenal wall have been reported.

**Table 1:** Related studies Case reports depicting various causes of OJ in patients with GAC [11-17] OJ: obstructive jaundice, GAC: gastric adenocarcinoma

Serial number	Authors	Study description
1	Tokoro et al. [11]	OJ in an operated case of GAC secondary to pancreatic head recurrence
2	Kawaoka et al. [12]	OJ in an operated case of GAC due to duodenal recurrence
3	Satake et al. [13]	OJ secondary to a metastatic deposit of GAC to the bile duct
4	Shirakawa et al. [14]	OJ in an operated case of GAC due to metastasis to the extrahepatic bile duct
5	Gabata et al. [15]	OJ in advanced GAC with the presence of both hepatoduodenal lymphadenopathy and tumoral involvement of the bile duct wall
6	Shimizu et al. [16]	Hepatic hilar lymph nodes causing OJ in a patient previously operated on for GAC
7	Watanabe et al. [17]	OJ secondary to liver metastasis from GAC

Saida et al. reviewed 129 cases of advanced and recurrent gastric carcinoma, 18 of whom developed obstructive jaundice during the course of their disease [18]. They concluded that differentiated cancer tends to have an expansile growth and is more likely to cause obstructive jaundice due to lymphadenopathy, while an undifferentiated tumor tends to infiltrate and causes biliary obstruction due to diffuse spread. Our case, being an undifferentiated gastric adenocarcinoma, was in congruence with their study.

## Conclusions

Obstructive jaundice in an adult should be thoroughly evaluated to rule out malignant biliary obstruction, including one due to gastric adenocarcinoma. The presence of obstructive jaundice in a known case of gastric carcinoma should warrant a search for hepatoduodenal ligament lymphadenopathy, direct spread across the gastroduodenal junction, bile duct wall thickening, and liver metastasis. The pattern of spread in gastric carcinoma, expansile mass-like growth with lymphadenopathy versus diffuse infiltrative spread, may predict the histological grade with the latter being a worse grade.
